# An Intranasal Vaccine Based on Outer Membrane Vesicles Against SARS-CoV-2

**DOI:** 10.3389/fmicb.2021.752739

**Published:** 2021-11-05

**Authors:** Himadri B. Thapa, Anna M. Müller, Andrew Camilli, Stefan Schild

**Affiliations:** ^1^Institute of Molecular Biosciences, University of Graz, Graz, Austria; ^2^Department of Molecular Biology and Microbiology, Tufts University School of Medicine, Boston, MA, United States; ^3^BioTechMed-Graz, Graz, Austria; ^4^Field of Excellence Biohealth, University of Graz, Graz, Austria

**Keywords:** outer membrane vesicles, Spike protein, SARS-CoV-2, RBD, *Vibrio cholerae*, enterotoxigenic *Escherichia coli*, outer member vesicles (OMV)

## Abstract

The prevailing pandemic of SARS-CoV-2 highlights the desperate need of alternative vaccine-platforms, which are safe, effective, and can be modified to carry antigens of emerging pathogens. The current SARS-CoV-2 vaccines based on mRNA and adenoviral vector technology meet some of these criteria but still face limitations regarding administration route, mass production, stability, and storage. Herein, we introduce a novel SARS-CoV-2 vaccine candidate based on bacterial outer membrane vesicles (OMVs). *Vibrio cholerae* and enterotoxigenic *Escherichia coli* (ETEC) have been genetically modified to produce increased amounts of detoxified OMVs decorated with the receptor binding domain (RBD) of the SARS-CoV-2 Spike protein. Intranasal immunization with RBD-decorated OMVs induced not only a robust immune response against the bacterial outer membrane components but also detectable antibody titers against the Spike protein. Cell culture infection assays using a Spike-pseudotyped lentivirus confirmed the presence of SARS-CoV-2 neutralizing antibodies. Highest titers against the SARS-CoV-2 Spike protein and most potent neutralization activity were observed for an alternating immunization regimen using RBD-decorated OMVs from ETEC and *V. cholerae* in turn. These results highlight the versatile vaccine applications offered by OMVs *via* expression of heterologous antigens in the donor bacterium.

## Introduction

Vaccines were invented over 200 years ago, with the recombinant vaccine era beginning in 1981 (Plotkin, 2014). Despite this long experience, generation of effective vaccines against emerging pathogens remains exceedingly difficult and still retains a significant empirical component. The current pandemic of SARS-CoV-2 highlights the desperate need of versatile vaccine-platforms, which can be easily modified to carry antigens of emerging pathogens. Within a remarkable time-frame and substantial investment we now have a handful of available vaccines that elicit significant protection against COVID-19, and ongoing vaccination efforts are successful in many countries (Abdulla et al., 2021; Pormohammad et al., 2021). However, the vaccines still face limitations as they are currently relying on invasive immunization routes (intramuscular), depend on highly specialized custom-built production pipelines, and require a cold-chain. Hence, mass production and worldwide distribution will be a huge challenge. Thus, the current and future pandemic outbreaks still warrant that we take a broad approach in developing safe and effective vaccine candidates. Herein, we present an outer membrane vesicle (OMV)-based vaccine candidate that is decorated with the receptor binding domain (RBD) of the SARS-CoV-2 Spike protein.

The Spike protein is present in multiple copies as protruding homotrimeric spikes on the viral envelope and mediates binding to the human ACE2 receptor triggering cell entry (Bosch et al., 2003). Spike is processed into two subunits, S1 and S2, which remain associated. The S1 subunit has a compact C-terminal domain that is largely composed of the RBD, which includes the receptor binding motif (RBM) directly binding the human ACE2 receptor. Importantly, antibodies recognizing epitopes within the RBM, or regions of the RBD outside of the RBM, can effectively neutralize the virus (Cao et al., 2020; Grifoni et al., 2020; Yi et al., 2020). For these reasons, current vaccine designs are mainly focused on the RBD of the SARS-CoV-2 Spike protein.

Outer membrane vesicles, which are naturally released by gram-negative bacteria during growth, are comprised largely of outer membrane, lipids, lipopolysaccharide (LPS), integral membrane proteins, and lipoproteins. OMVs possess characteristics that make them ideal candidates as a vaccine platform (Kulp and Kuehn, 2010; van der Pol et al., 2015; Pathirana and Kaparakis-Liaskos, 2016). First, they have intrinsic immunostimulatory properties due to the presence of pathogen-associated molecular patterns (PAMPs), primarily lipid A, porins, and lipoproteins. Together these PAMPs stimulate multiple pathogen recognition receptors (PRRs) in and on host cells, which in turn stimulate innate and adaptive immune responses (Ellis and Kuehn, 2010). Second, the OMV diameter of 20–200 nm allows them to drain freely into lymph nodes to target locally residing immune cells (van der Pol et al., 2015; Pathirana and Kaparakis-Liaskos, 2016). Thus, using the intranasal route of immunization will likely elicit a mucosal immune response in the airways to OMV antigens. Third, OMV production strains can be genetically engineered to express and decorate their surface with antigens of interest (Gerritzen et al., 2017).

Among others, we have been studying OMVs as vaccine candidates against human mucosal pathogens of the intestinal and respiratory tracts, e.g., members of the Pasteurellaceae, pathogenic *Escherichia coli*, and *Vibrio cholerae* (Schild et al., 2008, 2009; Bishop et al., 2010; Roier et al., 2012, 2013; Leitner et al., 2013, 2015). Overall, our studies show that non-invasive intranasal immunization induces a specific, high-titer, protective antibody response in the murine model that is long-lasting. Genetic engineering of donor strains allowed a deeper characterization of OMVs derived from *V. cholerae* and enterotoxigenic *Escherichia coli* (ETEC). For example, genetic modification of lipid A resulted in less endotoxicity without diminishing the immunogenic potential (Leitner et al., 2013, 2015). Furthermore, both bacterial species have been successfully genetically engineered to produce OMVs loaded with antigens of interest (Leitner et al., 2015; Gnopo et al., 2017).

Herein, we have genetically engineered detoxified ETEC and *V. cholerae* strains with increased OMV production. Using a Lpp-OmpA’ fusion strategy, previously used to express proteins of interest on the surface of *E. coli* K-12 bacteria (Francisco et al., 1992; Stathopoulos et al., 1996; Daugherty et al., 1998; Earhart, 2000), OMVs released by *V. cholerae* and ETEC could be efficiently decorated with the C-terminal part of the SARS-CoV-2 Spike protein S1 containing the RBD. Mice immunized with OMVs decorated with Lpp-OmpA-RBD (LOR) fusion protein induced a robust immune response not only against the bacterial surface components, but also against the Spike protein. SARS-CoV-2 neutralizing antibodies were confirmed in cell culture infection assays using the lentiviral SARS-CoV-2 pseudovirus in combination with 293T cells engineered to express the SARS-CoV-2 receptor ACE2.

## Materials and Methods

### Bacterial Strains, Cell Lines and Growth Conditions

Bacterial strains, cell lines and plasmids used in this study are listed in Table 1; oligonucleotides are listed in Table 2. AC53, a spontaneous streptomycin (Sm)-resistant mutant of the clinical isolate E7946 (O1 El Tor Ogawa), or ETEC H10407-S, a Sm-resistant mutant of the clinical isolate H10407, were used as wild-type strains (V-WT and E-WT). *E. coli* strain DH5αλ*pir* and SM10λ*pir* were used for genetic manipulations. Unless stated otherwise, strains were cultivated in Lysogeny broth (LB) or on LB agar plates with aeration at 37°C. If required, antibiotics and other supplements were used in the following final concentrations: streptomycin (Sm), 100 μg/ml; ampicillin (Ap), 100 μg/ml or in combination with other antibiotics 50 μg/ml; kanamycin (Km), 50 μg/ml; IPTG, 0.1 mM; glucose (Gluc), 0.2%; and sucrose (Suc), 10%.

**TABLE 1:** Bacterial strains, cell lines and plasmids used in this study.

Strain or plasmid	Genotype/resistance/description	Reference
***E. coli***		
DH5αλpir	F^–^Φ80*ΔlacZ*Δ*M15*Δ(*argF lac*)*U169 deoR recA1 endA1 hsdR17* (r_K_^–^m_K_^+^) *supE44 thi-1 gyrA69 relA1, λpir*R6K, Ap^r^	Hanahan, 1983
SM10λpir	thi thr leu tonA lacY supE recA::RPA-2-Te::Mu λpirR6K, Km^r^	Miller and Mekalanos, 1988
E-WT	H10407-S, wild type ETEC strain; serotype O78:H11; CFA/I LT^+^ STh^+^ STp^+^, Sm^r^	Evans and Evans, 1973
EΔ*msbB*Δ*eltA*	H10407 Δ*msbB*Δ*eltA*; Sm^r^	Leitner et al., 2015
EΔΔΔ	H10407 Δ*msbB*Δ*eltA*Δ*ompA*, Sm^r^	This study
***V. cholerae***		
V-WT	AC53, wild type *V. cholerae* strain serogroup: O1; biotype: El Tor; serotype: Ogawa; spontaneous Sm^r^ mutant of E7946; clinical isolate from Bahrain 1978; *hapR*^+^, Sm^r^; used for previous immunization studies (Schild et al., 2008, 2009; Bishop et al., 2010, 2012; Leitner et al., 2013)	Miller et al., 1989
VΔ*msbB*Δ*ctxAB*	AC53 Δ*msbB*Δ*ctxAB*; Sm^r^	Leitner et al., 2015
VΔΔΔ	AC53 Δ*msbB*Δ*ctxAB*Δ*ompA*; Sm^r^	This study
***Cell lines***		
HEK 293T	Parent cell line for transfection	ATCC, CRL-3216
HEK 293T-ACE2	Expresses human ACE2 receptor for pseudotype lentivirus expressing SARS-CoV-2 Spike protein	Crawford et al., 2020
HEK 293T-lentivirus Spike	Transfected HEK 293T cell line for making pseudotype lentivirus expressing SARS-CoV-2 Spike	This study
***Plasmids***		
p	pMMB67EH, IncQ broad-host-range cloning vector, IPTG-inducible, Ap^r^	Morales et al., 1991
pCVD442	ori6K mobRP4 *sacB*, Ap^r^	Donnenberg and Kaper, 1991
pΔompA-E	pCVD442 with up- and downstream fragments of *ompA* amplified from E-WT, Ap^r^	This study
pΔompA-V	pCVD442 with up- and downstream fragments of *V. cholerae ompA*, Ap^r^	Song et al., 2008
pMK-E-LOR	Standard cloning vector (Thermo Fisher Scientific) containing the Lpp-OmpA-RBD fusion construct codon optimized for *E. coli*, Km^r^	This study
pMK-V-LOR	Standard cloning vector (Thermo Fisher Scientific) containing the Lpp-OmpA-RBD fusion construct codon optimized for *V. cholerae*, Km^r^	This study
pLOR-E	pMMB67EH containing the Lpp-OmpA-RBD fusion construct codon optimized for *E. coli*, Ap^r^	This study
pLOR-V	pMMB67EH containing the Lpp-OmpA-RBD fusion construct codon optimized for *V. cholerae*, Ap^r^	This study
pHAGE2-CMV-luciferase-IRES-ZsGreen	For making pseudotype lentivirus host cell line, Ap^r^	Crawford et al., 2020
pHDM-Spike	For making pseudotype lentivirus host cell line, Ap^r^	Crawford et al., 2020
pHDM-Hgpm2	For making pseudotype lentivirus host cell line, Ap^r^	Crawford et al., 2020
pHDM-tat1b	For making pseudotype lentivirus host cell line, Ap^r^	Crawford et al., 2020
pRC-CMV-rev1b	For making pseudotype lentivirus host cell line, Ap^r^	Crawford et al., 2020

**TABLE 2:** Oligonucleotides used in this study.

Primer name	Sequence (5′ to 3′)^a^
ompA_E_SacI_1	AAAGAGCTCCGTGTCGTCAACGGTCAGG
ompA_E_EcoRI_2	TGAATTCTTTTTGCGCCTCGTTATCATC
ompA_E_EcoRI_3	TTTGAATTCGTTCTCGTCTGGTAGAAAAAC
ompA_E_XbaI_4	AAATCTAGACAGCAGTGTACGCAAAGAGA
LOR_E_1	ATCGGTAGAGTTAATATTGAGCAG
LOR_E_BamHI_2	TATGGATCCTTATTTGTCATCGTCGTCCTTGTAG
LOR_V_1	TCAGCGTATAACTCTCGACAATAAT
LOR_V_BamHI_2	AAAGGATCCTTATTTGTCATCGTCGTCCTTGT

*^*a*^Restriction sites are underlined.*

### Construction of Deletion Mutants and Expression Plasmids

The isolation of chromosomal DNA, PCR reactions, the purification of plasmids or PCR products, the construction of suicide and expression plasmids as well as the subsequent generation of deletion mutants were carried out as described previously using derivatives of pCVD442 or pMMB67EH (Seper et al., 2011; Leitner et al., 2015; Pressler et al., 2016). Qiagen plasmid kits were used for isolation of plasmid DNA; QIAquick^®^ Gel extraction and QIAquick^®^ PCR Purification kits (Qiagen) were used for purifying DNA fragments. PCR reactions for subcloning were carried out using the Q5^®^ High-Fidelity DNA Polymerase (NEB), while Taq DNA Polymerase (NEB) was used for all other PCRs. Construction of *ompA* in-frame deletion mutants in *V. cholerae* and ETEC were carried out as described by Donnenberg and Kaper (1991) using derivatives of pCVD442, i.e., pΔompA-V or pΔompA-E. The suicide vector pΔompA-V was already available from a previous study (Song et al., 2008). For construction of pΔompA-E, ∼800 bp PCR fragments located up- and downstream of the *ompA* were amplified using the oligonucleotide pairs ompA_E_SacI_1 and ompA_E_EcoRI_2 as well as ompA_E_EcoRI_3 and ompA_E_XbaI_4 with chromosomal DNA from E-WT as template (Table 2). After digestion of the PCR fragments with the appropriate restriction enzyme (NEB) indicated by the name of the oligonucleotide, they were ligated into pCVD442, which was digested with the appropriate restriction enzymes. Unless noted otherwise, ligation products were transformed into DH5αλpir and ApR colonies were characterized for the correct constructs by PCR. To obtain deletion strains, generated derivatives of pCVD442 were transformed into *E. coli* Sm10λpir and conjugated into *V. cholerae* or ETEC. Exconjugants were purified by Sm^R^/Ap^R^ selection. Sucrose selection was used to obtain Ap^S^ colonies and chromosomal deletions were confirmed by PCR, respectively.

The Lpp-OmpA-RBD*^*Vch*^* (LOR*^*Vch*^*) and Lpp-OmpA-RBD^ETEC^ (LOR^ETEC^) fusion proteins were designed *in silico* and the corresponding sequences including a 5′ untranslated region harboring a unique KpnI restriction site and an optimal Shine-Dalgarno sequence (Supplementary Figure 2) were synthesized and subcloned into the standard vector system pMK by the GeneArt Gene Synthesis platform (Thermo Fisher Scientific). Thus, constructs were provided as pMK-V-LOR and pMK-E-LOR. Finally, the expression plasmid pLOR-V and pLOR-E were constructed using the oligonucleotides LOR_V_1 and LOR_V_BamHI_2 as well as LOR_E_1 and LOR_E_BamHI_2 for amplifying the fusion construct using the ordered plasmids as template. This added a C-terminal FLAG-tag epitope to the constructs. The resulting PCR fragments were purified, digested with KpnI and BamHI, and ligated into pMMB67EH, which has been digested with the same enzymes. Ligation products were transformed into DH5αλpir and ApR colonies were characterized for the correct constructs by PCR.

### Preparation of Outer Membrane Vesicles

*Vibrio cholerae* as well as ETEC OMVs were isolated as previously described (Schild et al., 2008; Leitner et al., 2013, 2015). Briefly, overnight cultures grown in LB medium of the respective strains were diluted (1:100) in fresh LB and cultivated overnight at 24°C and 180 rpm. The cells were then removed from the supernatant by centrifugation (9,500 × *g*, 20 min, 4°C) and subsequent sterile filtration (0.22 μm). The OMVs present in the supernatant were pelleted through subsequent ultracentrifugation (150,000 × *g*, 4°C, 4 h) and resuspended in saline to generate a 1000-fold concentrated OMV suspension compared to the original filter-sterilized supernatant. Protein concentration was determined using Bradford assay (Bio-Rad Laboratories, Protein Assay Dye Reagent) according to the manufacturer’s manual. To quantify the LPS content of OMVs, purpald assays were performed as described previously using 3-deoxy-D-mannooctulosonic acid (Kdo) (Sigma-Aldrich) as a standard (Roier et al., 2016).

### Size Measurement

To estimate the size distributions of the isolated OMVs, dynamic light scattering (DLS) was carried out using Zetasizer Nano ZS90 (Malvern, United Kingdom) as previously described (Zingl et al., 2021b). Samples were diluted 1:1000 in saline and processed at 25°C under standard settings [Dispersant Refractive Index = 1.331, viscosity (cP) = 0.89]. Three measurements were performed using a measurement angle of 173° (backscatter), auto measurement duration, and “seek for optimal position” as positioning setting.

### Animals

Female BALB/c mice (Charles River Laboratories) were used in all experiments in accordance with the rules of the ethics committee at the University of Graz and the corresponding animal protocol, which has been approved by the federal ministry BMBWF (protocol: 39/12/75ex2017/18). Mice were housed with food and water *ad libitum* and monitored under the care of full-time staff. All animals were acclimated for 1 week before any procedures were carried out and were approximately 8 weeks old at the start of the immunization.

### Outer Membrane Vesicle Immunization Protocol

Eight-week-old female mice were intranasally immunized with OMVs (25 μg) in saline at days 0, 14, and 28 as described previously (Schild et al., 2008; Leitner et al., 2013). A group of sham (saline)-immunized control mice were housed in parallel with the immunized mice for the duration of each experiment. Overall two independent immunization rounds for each immunization group were performed with at least three mice per group. Comparison of the results from the independent immunization rounds revealed no differences in the induction of a humoral immune response in the respective immunization group.

### Preparation of Blood and Stool Samples

Blood samples as well as fecal pellets were collected from immunized and sham-immunized adult mice throughout the immunization study and processed as previously described to monitor the induced immune response (Schild et al., 2008, 2009). Briefly, blood samples were collected from immunized and sham immunized mice on days 0, 14, 28, 42, and 60. The collected blood was allowed to clot at room temperature (RT) for 30 min after which serum was isolated by removing the blood clot using a sterile toothpick followed by centrifugation (15 min, 1,000 × *g*). The supernatant representing the serum of each sample was removed, diluted threefold in PBS/sodium azide (0.05%), and subsequently stored at −80°C. In addition, aliquots of the sera collected on day 60 were stored at −80°C without any dilution or addition of sodium azide of to be used in cell culture neutralization assays. In case of stool samples, freshly voided fecal pellets collected on days 42 and 60 were vacuum-dried for 10 min before their weight was recorded. Immunoglobulins were extracted by adding 1 ml of extraction buffer [PBS, 0.01% sodium azide, 5% fetal calf serum, 1 tablet complete EDTA-free protease inhibitor cocktail (Roche) per ml] per 100 mg dry-weight feces. After vortexing the samples for 15 min at 4°C, solid material was separated by centrifugation (2 min, 13,000 × *g*) and the supernatants were stored at −80°C.

### Immunoprecipitation Using Outer Membrane Vesicles and Anti-receptor Binding Domain Antisera

For immunoprecipitation 9 μl of SARS-CoV-2 Spike Protein (RBD) Antibody (PA5-114451, Thermo Fisher Scientific) were mixed with 100 μg OMVs (protein equivalent determined by Bradford) and adjusted to final volume of 200 μl using saline. Antibodies were allowed to bind to the OMVs for 30 min at RT under gentle rocking before the OMVs were pelleted by centrifugation (75,600 × *g*, 4 h, 4°C). The OMV pellet was washed once with 1 ml saline and centrifuged again (75,600 × *g*, 2 h, 4°C) before the sample was finally resuspended in 40 μl saline and subjected to immunoblot analyses.

### Sodium Dodecyl Sulfate-Polyacrylamide Gel Electrophoresis and Immunoblot Analysis

To analyze the protein content of OMVs, the standard sodium dodecyl sulfate-polyacrylamide gel electrophoresis (SDS-PAGE) procedure in combination with 12% gels and the PageRuler Prestained Protein Ladder (Thermo Fisher Scientific) as a molecular mass standard were used. Approximately 7.5 μg of each sample was loaded and either stained according to the procedure of Kang et al. (2002) or transferred to a nitrocellulose membrane (Amersham) for immunoblot analysis, which was essentially performed as described previously (Roier et al., 2012). Either the anti-FLAG^®^ M2-HRP monoclonal antibody (A8592, Sigma Aldrich) was used as sole antibody or SARS-CoV-2 Spike Protein (RBD) Antibody (PA5-114451, Thermo Fisher Scientific) was used in combination with the HRP-linked anti-rabbit IgG (111-035-003, Jackson ImmunoResearch) were used as primary and secondary antibody, respectively.

### Enzyme-Linked Immunosorbent Assay

Temporal immune responses of different Igs, half-maximum total Ig titers and mucosal immune responses to VΔΔΔ or EΔΔΔ OMVs (5 μg/ml in PBS, pH 7.4) as well as the determination of the half-maximum total Ig titers to SARS-CoV-2 Spike Protein S1 (5 μg/ml in PBS, pH 7.4, RP-87679 Thermo Fisher Scientific) were carried out essentially as described previously (Roier et al., 2012; Leitner et al., 2013, 2015) using appropriate purified mouse Ig isotype standard (IgA, 553476; IgG1, 557273; or IgM, 550963, BD Biosciences) as well as horseradish peroxidase-conjugated goat anti-mouse antibodies (IgA, 62-6720; IgG1, A10551; IgM, 62-6820; IgG, IgM, IgA, PA1-84388, Invitrogen) in combination with the TMB peroxidase substrate reagent set (BioLegend). Optical densities were monitored at 450 nm with a FLUOstar Omega microplate reader (BMG Labtech). Starting dilutions of the mouse sera were 1:10 for quantification of the temporal immune responses and half-maximum total Ig titers against the Spike protein, 1:100 for quantification of the half-maximum total Ig titers against OMVs, or 1:400 for quantification of the temporal immune responses against OMVs. Starting dilutions of the fecal pellet extracts for quantification of the mucosal immune responses against OMVs were 1:10. If the highest sample concentration (sera or fecal pellet extracts) used did not yield any detectable absorption the value was set to the respective limit of detection of the assay stated in each figure legend.

### Spike Pseudotyped Lentivirus Particles

Plasmids pHAGE2-CMV-luciferase-IRES-ZsGreen, pHDM-Spike, pHDM-Hgpm2, pHDM-tat1b, and pRC-CMV-rev1b were transfected into HEK 293T cells as described (Crawford et al., 2020). The plasmids and HEK 239T-ACE2 cell line were obtained from BEI Resources (Manassas, VA, United States). Transfection was confirmed by scoring green fluorescence of the cells 24 h later. Viral particles were collected from cell culture supernatants after 48 h as described (Crawford et al., 2020) and were stored in single-use aliquots at −80°C. The titer of thawed virus stock was 1.5 × 10^5^ infectious units/ml.

### Serum Neutralization of Spike-Pseudotyped Lentivirus

Neutralization assays were performed essentially as recently described (Crawford et al., 2020). Briefly, serial dilutions of each mouse serum were made in duplicate in 96-well plates (12565501, Fisher Scientific) in Dulbecco’s Modified Eagle Medium (DMEM) (MT10013CV, Corning) supplemented with 10% heat-inactivated fetal bovine serum (16140063, Gibco), and penicillin (100 U/ml) and streptomycin (100 μg/ml) (15140148, Gibco). A volume of 150 μl DMEM containing 3.1 × 10^3^ infectious units of pseudotype virus was added to each well and incubated for 1.2 h at 37°C in a 5% CO_2_ incubator to allow antibodies to bind. This amount of virus gave a multiplicity of infection of approximately 0.08. Polybrene (TR1003E, Sigma) was added to each well to increase the efficiency of viral infection just before transferring the contents of each well to semi-confluent monolayers of HEK 293T-ACE2 cells in solid white 96-well plates (07-200-591, Fisher Scientific). The plates were incubated for 48 h at 37°C in a 5% CO_2_ incubator to allow for viral infection. Infection by the Spike-pseudotyped lentivirus expressing Luciferase-IRES-ZsGreen was quantified by measuring light production after removing spent medium and adding Bright-Glo Luciferase reagent (E2610, Promega). After mixing for 20 s by swirling, each plate was incubated in the dark for 2 min, followed by measuring relative light units (RLU) on a Synergy^TM^ HT Microplate Reader using Gen5 data analysis software (BioTek). Background was initially assayed from uninfected cells, virus in the absence of cells, or DMEM medium alone, and all were equivalent. Therefore, in subsequent experiments, each plate contained at least two DMEM alone wells. The maximum infection on each plate was measured by infecting at least four wells of HEK 293T-ACE2 cells with pseudotype virus that had not been neutralized with serum. The fraction infectivity was calculated by averaging technical replicates and dividing by the average of the maximum infectivity on that plate. The inhibitory concentration 50% (IC50) was calculated graphically.

### Data Presentation and Statistical Analysis

The data is generally presented as median with interquartile range as some data sets were not normally distributed. Consequently, data were analyzed using the Mann–Whitney *U* test or a Kruskal–Wallis test followed by *post hoc* Dunn’s multiple comparisons. Differences were considered significant at *p*-values of < 0.05. GraphPad Prism version 8 was used for all statistical analyses.

## Results

### Generation of *V. cholerae* and Enterotoxigenic *Escherichia coli* Outer Membrane Vesicles Decorated With the SARS-CoV-2 Receptor Binding Domain Antigen

We recently demonstrated that OMVs derived from ETEC or *V. cholerae* lacking one functional secondary lipid A acyltransferase, MsbB (also referred to as LpxN) retain their potential to induce a high-titer, protective immune response but show a significantly reduced pro-inflammatory cytokine induction suggesting lower endotoxicity (Leitner et al., 2013, 2015). Moreover, cholera toxin subunits A and B in *V. cholerae*, and the heat-labile enterotoxin catalytic A subunit in *E. coli* were deleted. Thus, presence of these secreted toxins in OMVs is precluded (Leitner et al., 2015). In addition to these modifications resulting in detoxified OMVs, we also deleted *ompA*, encoding the outer membrane protein OmpA, which crosslinks the periplasmic peptidoglycan and the outer membrane. Deletion of *ompA* has been reported to result in elevated OMV release in various gram-negative bacteria (Song et al., 2008; Schwechheimer and Kuehn, 2015). Concordantly, the engineered triple mutants VΔΔΔ and EΔΔΔ showed a significant three- to six-fold increase in OMV production compared to the parental WT strains (V-WT and E-WT), respectively (Supplementary Figure 1). Thus, the engineered triple mutants VΔΔΔ and EΔΔΔ are optimized *V. cholerae* and ETEC platform strains for production of OMVs to be used for vaccination.

**FIGURE 1:**
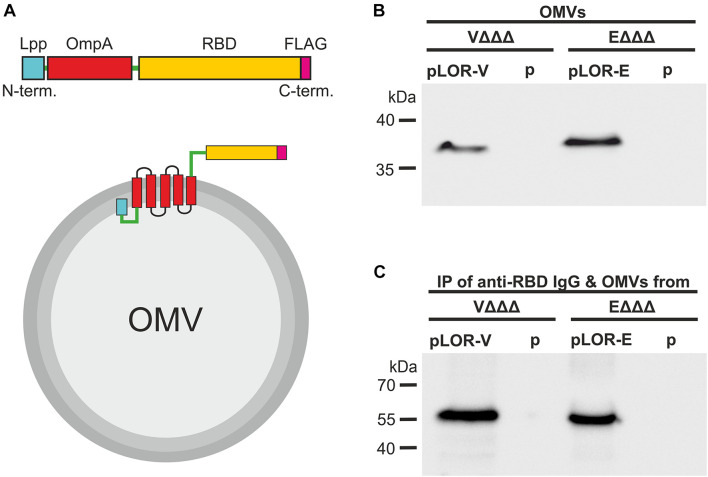
Decoration of OMVs with RBD antigens *via* a Lpp-OmpA fusion strategy. **(A)** Schematic design of the Lpp-OmpA-RBD (LOR) fusion constructs (top) and expected localization in the OMVs (bottom). Relevant parts of the LOR include the Lpp fragment (light blue), linkers (green), OmpA fragment (red), theSARS-CoV-2 RBD fragment (yellow), and the FLAG-tag (pink). Panel **(B)** shown is a representative immunoblot detecting the LOR fusion protein in OMVs derived from VΔΔΔ pLOR-V and VΔΔΔ p, EΔΔΔ pLOR-E, and EΔΔΔ p. The commercially available anti-RBD antisera specifically detecting the SARS-CoV-2 Spike protein was used for this immunoblot. A Kang-stained SDS gel executed in parallel served as loading control (Supplementary Figure 3). Panel **(C)** shown is a representative immunoblot detecting the heavy chain (approx. 55 kDa) of the commercially available anti-RBD antisera after an immunoprecipitation (IP) with OMVs derived from VΔΔΔ pLOR-V and VΔΔΔ p, EΔΔΔ pLOR-E, and EΔΔΔ p. OMVs of the respective strains were co-incubated with the anti-RBD antisera (IgG), subsequently pelleted and washed by centrifugation steps and finally separated by SDS-PAGE for immunoblot analyses (for details please see “Materials and Methods” section). The commercially available HRP-linked anti-rabbit antibody detecting the heavy chain of the anti-RBD IgG raised in rabbits was used for this immunoblot. A Kang-stained SDS gel executed in parallel served as loading control (Supplementary Figure 4). **(B,C)** Molecular mass standards (Prestained Protein Marker Broad Range–New England Biolab) are indicated on the left.

To ensure efficient translocation to the outer membrane and external exposure of the SARS-CoV-2 RBD domain, we adapted the Lpp-OmpA’ fusion strategy, which has been used previously to display a variety of heterologous proteins on the bacterial surface of *E. coli* (Francisco et al., 1992; Stathopoulos et al., 1996; Daugherty et al., 1998; Earhart, 2000). The general design of the LOR fusion constructs was similar for *V. cholerae* and ETEC (Figure 1A). The LOR fusion constructs consist of a N-terminal signal sequence and next nine amino acids of the major outer membrane lipoprotein (Lpp), followed by a two-amino-acid linker, and then approximately 110 amino acids of OmpA, i.e., amino acids 46–146 of ETEC and amino acids 51–158 of *V. cholerae* OmpA. The Lpp fragment directs the fusion protein to the outer membrane, with the acylated N-terminal end being anchored in the inner leaflet of the outer membrane. The OmpA fragment contains five membrane-spanning segments with the C-terminus residing externally (Francisco et al., 1992). *Via* an additional four-amino-acid linker the heterologous peptide consisting of the entire RBM and the majority of the RBD of SARS-CoV-2 is fused to the C-terminal end. As Lpp and OmpA of *V. cholerae* and ETEC share only 38% identity, the amino acid sequence as well as the SARS-CoV-2 Spike protein is neither optimal for *E. coli* nor *V. cholerae*; we have used species-specific fusion constructs using the individual *lpp* and *ompA* sequences as well as codon optimized sequences (obtained *via* the IDT codon optimization tool) of the SARS-CoV-2 RBD for each species, namely, LOR-V and LOR-E, respectively (Supplementary Figure 2). Along the design, a unique KpnI restriction site for subsequent cloning into expression vectors and an optimized Shine Dalgarno sequence ensuring high translation efficiency were included in the 5′ untranslated region. Synthesized fragments were PCR-amplified, which added a C-terminal FLAG-tag epitope to the fusion proteins, and subcloned into the IPTG-inducible broad range vector pMMB67EH (see Supplementary Figure 2 and “Materials and Methods” section for details) resulting in the LOR fusion expression constructs pLOR-V and pLOR-E.

Expression of the LOR fusion proteins in VΔΔΔ and EΔΔΔ should result in their localization on the bacterial cell surface and therefore also on the surface of OMVs, which bud from the outer membrane. Successful localization of LOR in OMVs was confirmed by immunoblot analyses (Figure 1B and Supplementary Figure 3). Detection of LOR fusion proteins with anti-FLAG and anti-RBD antibodies showed specific bands with the expected molecular weight of approximately 36 kDa for *V. cholerae* and 38 kDa for ETEC for the OMVs derived from VΔΔΔ pLOR-V and EΔΔΔ pLOR-E, but not for OMVs isolated from strains harboring the empty vector (VΔΔΔ p and EΔΔΔ p). Thus, OMVs of both species can be decorated with significant amounts of the LOR. Furthermore, outward exposure of RBD part on OMVs was confirmed *via* an immunoprecipitation assay using OMVs derived from VΔΔΔ pLOR-V, VΔΔΔ p, EΔΔΔ pLOR-E, and EΔΔΔ p as antigen in combination with anti-RBD antibodies. After incubation of OMVs and anti-RBD antibodies, samples were subjected to centrifugation and washing steps to purify OMVs. Finally, immunoblot analyses with the immunoprecipitated samples were performed to detect anti-RBD antibody (raised in rabbits) bound to the OMV samples using an HRP-conjugated anti-rabbit antisera (Figure 1C). A Kang-stained gel executed in parallel served as a loading control (Supplementary Figure 4). Heavy chains of rabbit IgG, indicating bound anti-RBD antibodies to OMVs, were detected at the expected molecular weight of approximately 55 kDa in immunoprecipitations using OMVs derived from VΔΔΔ pLOR-V and EΔΔΔ pLOR-E, but not for OMVs isolated from strains harboring the empty vector (VΔΔΔ p and EΔΔΔ p). The accessibility of the LOR fusion proteins on OMVs by the anti-RBD antibody strongly suggests that the RBD portion is residing externally on the surface of the OMVs.

Finally, we determined the size distribution and quantified the biomass of OMVs liberated by the parental WT strains (V-WT and E-WT), the triple mutants (VΔΔΔ and EΔΔΔ) as well as the donor strains for the OMV-vaccine candidates used herein (i.e., VΔΔΔ p, EΔΔΔ p, VΔΔΔ pLOR-V, and EΔΔΔ pLOR-E) (Supplementary Figure 5). These analyses confirmed the increased OMV biomass amounts liberated by the triple mutants in both species.

### Intranasal Immunization of Lpp-OmpA-Receptor Binding Domain-Outer Membrane Vesicles Decorated With SARS-CoV-2 Receptor Binding Domain Induces a Robust Immune Response Against the Spike Protein

**FIGURE 2:**
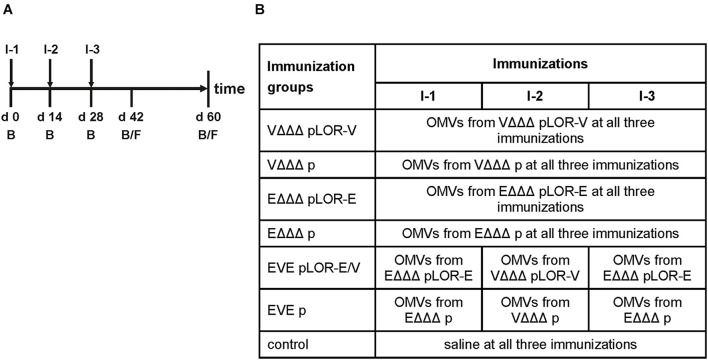
Design of the immunization study. **(A)** Timeline is given by a horizontal arrow from left to the right. Each vertical bar marks the day of a bleed (B) or fecal pellet (F) collection. Mice received three intranasal immunizations starting with the initial immunization on day 0, followed by boosts on days 14 and 28. Immunizations are indicated by I-1, I-2, and I-3 and are highlighted by vertical arrows. **(B)** The study comprised seven different immunization groups (*n* = 7 for each group) being named after the donor strains of the OMVs used for immunization, i.e., VΔΔΔ pLOR-V, VΔΔΔ p, EΔΔΔ pLOR-E, EΔΔΔ p, EVE pLOR-E/V, and EVE p. Immunization groups VΔΔΔ pLOR-V, VΔΔΔ p, EΔΔΔ pLOR-E, or EΔΔΔ p received the OMVs of the respective donor strain in all three immunizations (I-1 to I-3). The immunization groups EVE pLOR-E/V and EVE p were immunized with alternating regimen and received OMVs from EΔΔΔ pLOR-E or EΔΔΔ p on days 0 (I-1) and 28 (I-3) as well as OMVs from VΔΔΔ pLOR-V or VΔΔΔ p on day 14 (I-2). A sham-immunized group receiving saline at days 0, 14, and 28 served as control.

In order to investigate the immune response upon immunization with LOR-decorated OMVs (LOR-OMVs), we intranasally immunized mice with OMVs derived from VΔΔΔ pLOR-V, VΔΔΔ p, EΔΔΔ pLOR-E, and EΔΔΔ p according to our standard immunization protocol (Schild et al., 2008; Figure 2A). Briefly, mice were intranasally immunized on days 0, 14, and 28 receiving 25 μg OMVs (protein equivalent) per immunization. In total, seven different immunization groups were tested (Figure 2B). Four groups received the same OMVs at days 0, 14, and 28 and were therefore only immunized with OMVs from either VΔΔΔ pLOR-V, VΔΔΔ p, EΔΔΔ pLOR-E, or EΔΔΔ p. Two groups (EVE pLOR-E/V and EVE p) were immunized with alternating regimen and received OMVs from EΔΔΔ pLOR-E on days 0 and 28 as well as OMVs from VΔΔΔ pLOR-V on day 14 (EVE pLOR-E/V) or EΔΔΔ p on days 0 and 28 as well as VΔΔΔ p on day 14 (EVE p). A change of the OMV donor species between the immunizations was thought to specifically boost the immune response against the RBD of SARS-CoV-2 being the common antigen on OMVs derived from VΔΔΔ pLOR-V and EΔΔΔ pLOR-E. Finally, a sham-immunized group receiving saline at days 0, 14, and 28 served as control.

**FIGURE 3:**
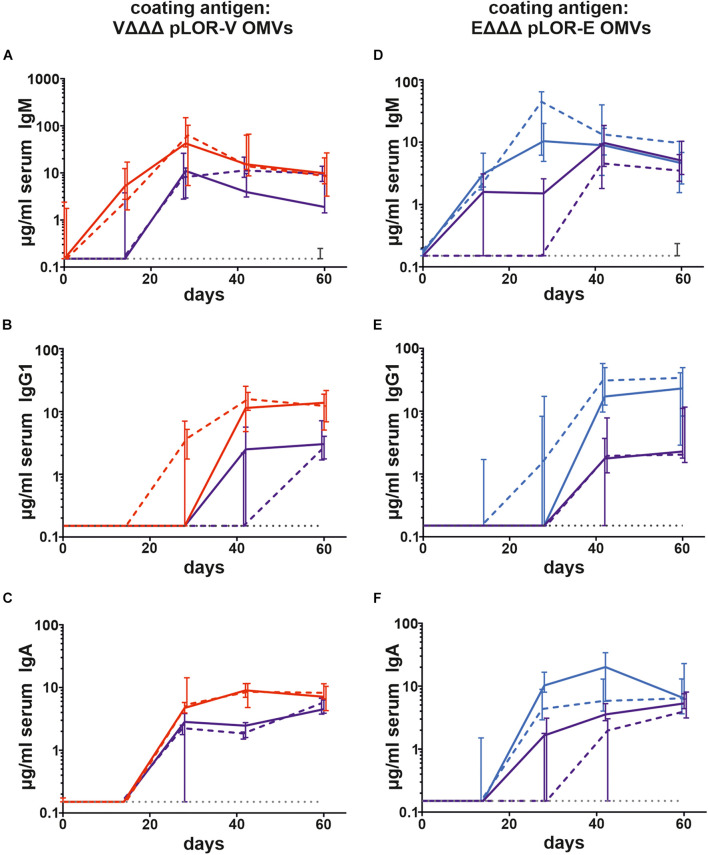
Temporal immune responses to *V. cholerae* and ETEC OMVs. Shown are the median titers over time of IgM **(A,D)**, IgG1 **(B,E)**, and IgA **(C,F)** antibodies to OMVs derived from VΔΔΔ pLOR-V **(A–C)** and EΔΔΔ pLOR-E **(D–F)** in sera from mice intranasally immunized with OMVs derived from VΔΔΔ pLOR-V (red solid line), VΔΔΔ p (red dashed line), EΔΔΔ pLOR-E (blue solid line), EΔΔΔ p (blue dashed line), and from the sham-immunized control group (gray dotted line). Moreover, two groups were intranasally immunized with alternating regimen and received OMVs from EΔΔΔ pLOR-E on days 0 and 28 as well as OMVs from VΔΔΔ pLOR-V on day 14 (EVE pLOR-E/V, purple solid line) or OMVs from EΔΔΔ p on days 0 and 28 as well as VΔΔΔ p on day 14 (EVE p, purple dashed line). Samples which did not yield in any detectable signal at their highest concentration were set to the limit of detection (0.15 μg/ml). The error bars indicate the interquartile range of each data set for each time point (*n* = 7 for each group).

To monitor the immune responses in sera of immunized and sham-immunized control mice by enzyme-linked immunosorbent assay (ELISA), OMVs derived from VΔΔΔ pLOR-V or EΔΔΔ pLOR-E were used as coating material allowing the detection of temporal IgM, IgG1, and IgA responses to the respective OMVs (Figure 3). Herein we focused on IgG1 as previous studies revealed IgG1 to be the dominant IgG subclass induced after intranasal immunization with OMVs (Schild et al., 2008, 2009; Bishop et al., 2010; Leitner et al., 2013, 2015). As reported earlier, immunization with *V. cholerae* or ETEC OMVs induces a species-specific immune response without any significant cross-reactivity (Leitner et al., 2015). Thus, groups immunized with OMVs solely derived from one species were only tested for immune responses against OMVs derived from the same species, while immunization groups with alternating regimen and the sham-immunized control group were tested for immune responses against both, OMVs derived from *V. cholerae* and ETEC. The antibody titers of the sham-immunized control group were determined for days 0 and 60 and remained below the limit of detection or at very low levels for both time points against OMVs derived from *V. cholerae* and ETEC, respectively. In general, the induction of an immune response was characterized by an Ig titer increase during the vaccination period. Thus, IgM titers peaked at days 28 or 42, while IgG and IgA were slightly delayed and peaked on days 42 or 60. Not surprisingly, Ig titers of the immunization groups receiving the same OMVs at all three immunization were overall higher and reached the Ig peak earlier than the immunization groups with alternating regimen. Importantly, IgG1 and IgA titers remained stable or even increased between days 42 and 60, which indicates the induction of a robust immune response until day 60, the designated endpoint of this study.

**FIGURE 4:**
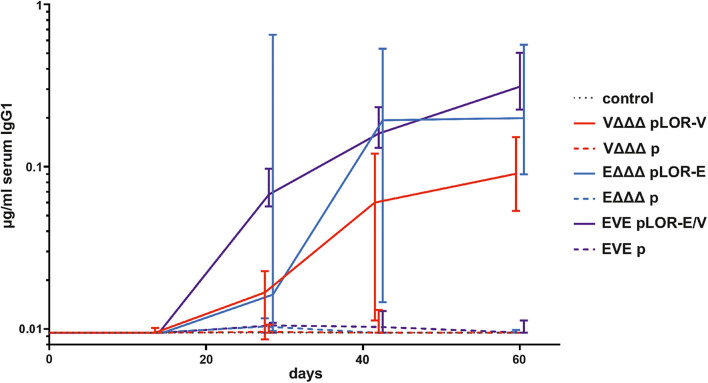
Temporal immune responses against the SARS-CoV-2 Spike protein. Shown are the median titers over time of IgG1 antibodies to SARS-CoV-2 Spike protein in sera from mice intranasally immunized with OMVs derived from VΔΔΔ pLOR-V (red solid line), VΔΔΔ p (red dashed line), EΔΔΔ pLOR-E (blue solid line), EΔΔΔ p (blue dashed line), and from the sham-immunized control group (gray dotted line). Moreover, two groups were intranasally immunized with alternating regimen and received OMVs from EΔΔΔ pLOR-E on days 0 and 28 as well as OMVs from VΔΔΔ pLOR-V on day 14 (EVE pLOR-E/V, purple solid line) or OMVs from EΔΔΔ p on days 0 and 28 as well as VΔΔΔ p on day 14 (EVE p, purple dashed line). Samples which did not yield in any detectable signal at their highest concentration were set to the limit of detection (0.01 μg/ml). The error bars indicate the interquartile range of each data set for each time point (*n* = 7 for each group).

Next, we evaluated the induction of a humoral immune response against the SARS-CoV-2 Spike protein. Based on the high IgG1 titers in serum samples we focused on this isotype to analyze the temporal immune response against the Spike protein (Figure 4). IgG1 titers of the sham-immunized control group as well as the immunization groups VΔΔΔ p, EΔΔΔ p, and EVE p remained below the limit of detection or at very low levels at all time points, respectively. In contrast, all three immunization groups receiving OMVs decorated with LOR, i.e., VΔΔΔ pLOR-V, EΔΔΔ pLOR-E, and EVE pLOR-E/V showed detectable IgG1 titers against the Spike protein from days 28 onward, which peaked on days 42 or 60. We also tried to determine serum IgA responses against the Spike protein, but IgA titers were overall lower compared to the IgG1 titers and remained below the limit of detection (0.04 μg/μl) even for several serum samples of the immunization groups receiving OMVs decorated with LOR. Unfortunately, this analysis was hampered by observable cross-reactivity of the secondary antibody with serum components or the Spike protein combined with relatively low IgA titers in the samples.

**FIGURE 5:**
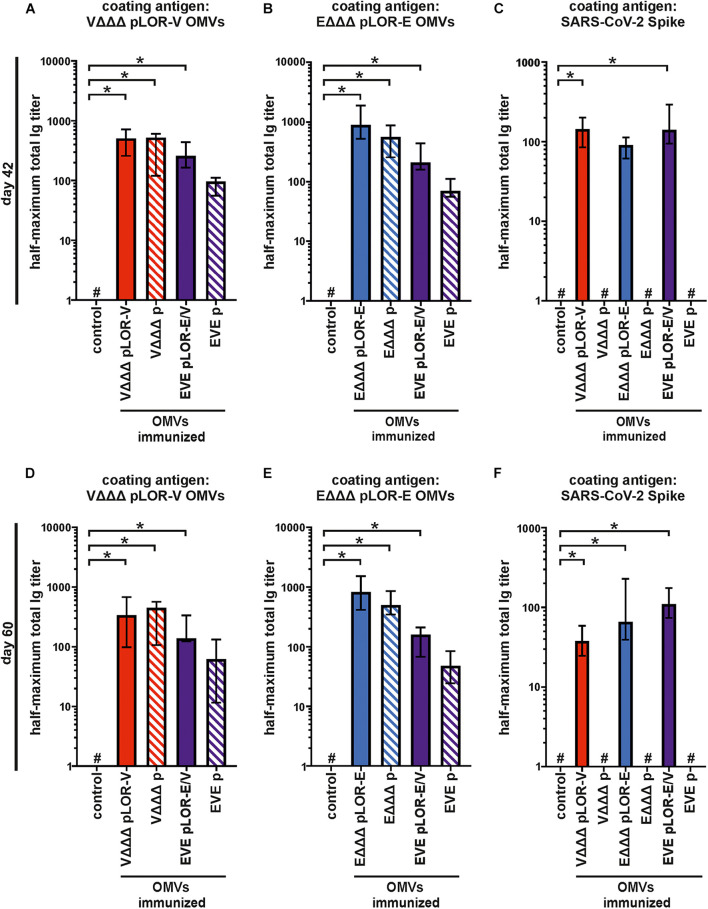
Quantification of the total immune response against *V. cholerae*, ETEC and SARS-CoV-2 Spike protein induced in OMV-immunized mice. Shown are median half-maximum total Ig titers to OMVs derived from VΔΔΔ pLOR-V **(A,D)** and EΔΔΔ pLOR-E **(B,E)** as well as to SARS-CoV-2 Spike protein **(C,F)** in sera collected at day 42 **(A–C)** or day 60 **(D–F)** from mice of the respective immunization groups as indicated (*n* = 7 for each group). The error bars indicate the interquartile range of each data set and the hash key that the result of the data set was below the limit of detection, which was set to 1. Significant differences to the sham-immunized control group are marked by asterisks (*p* < 0.05; Kruskal–Wallis test and *post hoc* Dunn’s multiple comparisons).

To further characterize the induced humoral immune response, sera from days 42 and 60 were used to determine the half-maximum total Ig titers against OMVs derived from VΔΔΔ pLOR-V or EΔΔΔ pLOR-E as well as against SARS-CoV-2 Spike protein (Figure 5). In comparison to the sham-immunized control group the immunization groups receiving OMVs showed a marked increase in total Ig titers for both time points against the respective OMVs derived from VΔΔΔ pLOR-V or EΔΔΔ pLOR-E (Figures 5A,B,D,E). Similar to the temporal immune response patterns, the OMV-specific responses were slightly lower in the immunization groups with alternating regimen (EVE pLOR-E/V and EVE p) compared to the immunization groups receiving the same OMVs at all three immunizations. In comparison to the sham-immunized control group, a significant increase of the total Ig titers against the Spike protein was observed at day 42 for the VΔΔΔ pLOR-V and EVE pLOR-E/V immunization group as well as for all immunization groups receiving OMVs decorated with LOR (i.e., VΔΔΔ pLOR-V, EΔΔΔ pLOR-E and EVE pLOR-E/V) at day 60 (Figures 5C,F). In contrast, to the OMV-specific immune response, the EVE pLOR-E/V immunization group showed slightly, but not significantly higher Ig titers against the Spike protein than the VΔΔΔ pLOR-V and EΔΔΔ pLOR-E groups. For both time points tested, the immune response against the Spike protein remained undetectable or at low levels for immunization groups receiving OMVs from strains harboring the empty vector (VΔΔΔ p, EΔΔΔ p and EVE p). In general, isotype-specific and total Ig titers did not massively drop between days 42 and 60 in the respective immunization groups suggesting the induction of a stable immune response (Figures 3–5).

**FIGURE 6:**
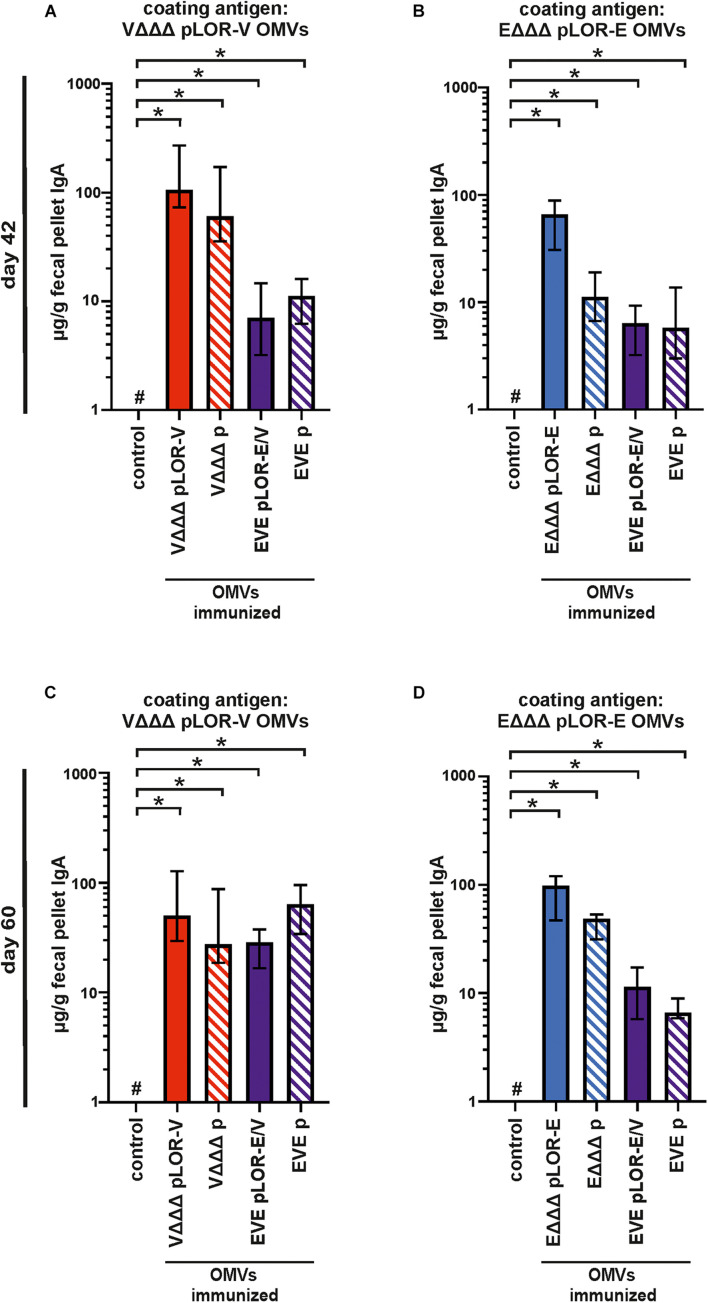
Mucosal immune responses induced in OMV-immunized mice. Shown are median IgA titers to OMVs derived from VΔΔΔ pLOR-V **(A,C)** and EΔΔΔ pLOR-E **(B,D)** extracted from fecal pellets collected at day 42 **(A,B)** or day 60 **(C,D)** from the respective immunization groups as indicated (*n* = 7 for each group). The error bars indicate the interquartile range of each data set and the hash key that the result of the data set was below the limit of detection (1 μg/g). Significant differences to the sham-immunized control group are marked by asterisks (*p* < 0.05; Kruskal–Wallis test and *post hoc* Dunn’s multiple comparisons).

In addition to the humoral immune responses, we also analyzed the induced mucosal immune responses by measuring the secretory IgA titers in fecal pellet extracts collected at days 42 and 60 against OMVs derived from VΔΔΔ pLOR-V or EΔΔΔ pLOR-E (Figures 6A–D). Fecal pellet collection was preferred over other methods (e.g., saliva collection or lung lavage) as it seems less stressful and allowed us to keep the mice alive for later time points. Furthermore, it has been shown in earlier studies that secretory IgA antibodies reflecting the mucosal immune response can also be found in feces upon OMV immunization and that IgA titers in feces correlate by trend with those found in the respiratory tract, e.g., saliva (Vetvik et al., 1998; Hirano et al., 2006; Schild et al., 2009).

Generally, the mucosal immune response on days 42 and 60 showed a similar pattern as the half-maximum total Ig titers. All immunization groups receiving OMVs showed a significant increase in secretory IgA titers against the respective OMVs compared to the sham-immunized control group (Figures 6A–D). In majority of the cases, the secretory IgA titers against OMVs were slightly lower in immunization groups with alternating regimen (EVE pLOR-E/V and EVE p) compared to the immunization groups receiving the same OMVs at all three immunizations. We also tried to determine secretory IgA levels against the Spike protein in fecal pellet extracts, but for the majority of samples the titers remained below or close to the limit of detection (1 μg/g). As described above, this analysis was hampered by observable cross-reactivity of the secondary antibody with components of the fecal pellet extract or the Spike protein combined with relatively low concentration of mucosal IgA in the samples.

### Immunization With *V. cholerae* and Enterotoxigenic *Escherichia coli* Lpp-OmpA-Receptor Binding Domain-Outer Membrane Vesicles Elicits Neutralizing Antibody Titers

**FIGURE 7:**
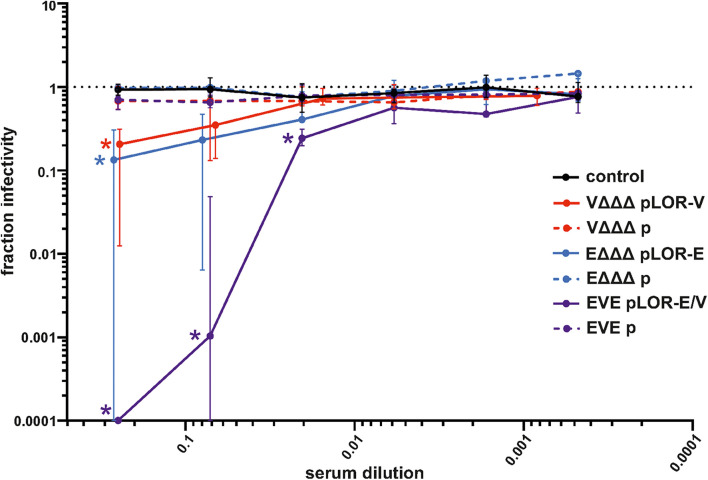
Neutralization assays with sera from OMV-immunized mice. Shown is the median fraction of infectivity using a Spike-pseudotyped lentivirus neutralization assay in combination with sera collected at day 60 from mice of the respective immunization groups as indicated (*n* = 7 for each group). Starting with a 1:4 dilution, 3.5-fold serial dilutions of each sera were tested. The error bars indicate the interquartile range of each data set. The limit of detection in these assays was a fraction of infectivity = 0.0001. Overlapping median values and error bars were slightly nudged on the *x*-axis to allow better visualization. Significant reductions of infectivity compared to the sham-immunized control group are marked by asterisks in the color of the respective data set and were observed for the following data sets: VΔΔΔ pLOR-V and EΔΔΔ pLOR-E for the 1:4 serum dilution as well as EVE pLOR-E/V for the 1:4, 1:14, and 1:49 dilution (*p* < 0.05; Kruskal–Wallis test and *post hoc* Dunn’s multiple comparisons).

To determine if the vaccines were capable of eliciting neutralizing antibodies, a Spike-pseudotyped lentivirus neutralization assay was used as previously described (Crawford et al., 2020). Functionality of the assay was confirmed by using commercially available anti-RBD antisera as positive control and PBS as solvent (negative) control, which revealed a robust neutralizing activity of the anti-RBD antisera, but not for the solvent control (Supplementary Figure 6). Briefly, a pseudotype lentivirus expressing the SARS-CoV-2 Spike protein on its envelope and, upon infection, luciferase in the host cell cytoplasm, was incubated with each mouse serum to allow antibody binding, followed by infection of HEK 293T cells expressing the ACE2 receptor. The fraction infectivity was determined by measuring RLU of the monolayers. An amount of virus was used to achieve a signal >1000 above background. Sera from the sham-immunized control group and immunization groups receiving OMVs from strains harboring the empty vector (VΔΔΔ p, EΔΔΔ p, and EVE p) showed no measurable neutralization activity. Using the highest sera concentration, i.e., 1:4 dilution, a significant neutralizing activity was observed for all three immunization groups receiving OMVs decorated with LOR, i.e., VΔΔΔ pLOR-V, EΔΔΔ pLOR-E and EVE pLOR-E/V, compared to the sham-immunized control group (Figure 7). In case of sera from the EVE pLOR-E/V group a significant neutralizing activity was also detectable for following two dilutions (1:14 and 1:49). The IC50 for the serum from the immunization group with alternating regimen (EVE pLOR-E/V) was approximately 1:142, which was about fourfold higher than for the repeating regimens (VΔΔΔ pLOR-V and EΔΔΔ pLOR-E).

## Discussion

In this study, we focused on the development of a mucosally delivered, vesicle-based, subunit vaccine, which can stimulate a robust immune response directed against the SARS-CoV-2 Spike protein. Therefore, we introduce a bacterial OMV decoration strategy utilizing Lpp-OmpA’ fusions. OMVs derived from *V. cholerae* and ETEC have been chosen as platform as both are highly immunogenic and have been successfully used in previous immunization studies (Schild et al., 2008, 2009; Bishop et al., 2010; Leitner et al., 2013, 2015). Parental strains of *V. cholerae* and ETEC have been optimized to exhibit increased OMV release, lack the enterotoxins, and show reduced LPS reactivity. The genetically engineered OMV production strains were successfully used for heterologous antigen expression to generate detoxified OMVs decorated with the RBD portion of the SARS-CoV-2 Spike protein. In general, intranasal immunization with OMVs induced a robust immune response against surface structures of the respective donor bacteria, but only mice immunized with RBD-decorated OMVs induced a significant Spike protein-specific immune response. Concordantly, neutralizing activity was only observed for sera from mice immunized with RBD-decorated OMVs. The results from the Spike-pseudotyped lentivirus neutralization assay indicate the induction of a neutralizing-antibody response upon intranasal immunization with RBD-decorated OMVs.

An interesting and potentially important finding was the increased efficacy of the alternating immunization regimen using RBD-decorated OMVs derived from ETEC and from *V. cholerae* (the EVE pLOR-E/V group). This group not only showed the highest titers against the Spike protein, but sera from that group also exhibited the strongest potency for neutralization in the cell culture infection assay. Thus, a change of the OMV donor species between immunization can specifically boost the immune response against the heterologously expressed common antigen of interest, i.e., RBD of the Spike protein. A similar approach was successfully applied along the development of the recombinant adenovirus-based vaccine (Sputnik V), which uses two different adenovirus vectors, with rAd26 in the first and rAd5 in the second immunization dose, respectively (Logunov et al., 2021). It should be noted that the alternating regimen reduces the immunization with OMVs derived from ETEC to two doses and OMVs derived from *V. cholerae* to one dose. Thus, it is not surprising that the immune response against the OMVs themselves is lowest in these groups compared to the groups receiving the same OMV type in all three immunizations.

The study raises several questions to be addressed in future studies, such as the longevity of the immune response, the optimal OMVs amount per immunization dose, and number of booster immunizations. Previous reports demonstrated that a two-dose immunization schedule with *V. cholerae* OMVs is sufficient to induce a long-lasting, protective immune response against the bacterial pathogen (Bishop et al., 2010). Thus, three immunizations might not even be required. Along this first report of our OMV-based SARS-CoV-2 vaccine candidate we focused on the original Wuhan Hu-1 isolate. Future studies need to address the neutralization activity against epidemiologically relevant SARS-CoV-2 variants with RBM mutations, which have been emerging in the last months. However, even if a reduced efficacy would be observed, the fusion strategy presented herein is adaptable to decorate OMVs with appropriate antigens upon emergence of new viral variants.

Some OMV-based vaccine technologies purify the OMVs and the heterologous antigens separately in a first step to subsequently coat or load the vesicles in a second step (Li and Liu, 2020; Zingl et al., 2021a). Expression of the heterologous antigen directly in the bacterial OMV donor species simplifies the production process and may reduce costs. Once purified, the decorated OMVs could be used for immunization without any further manipulation. However, it is likely that host-specific post-translational modifications are lacking along the bacterial expression system., e.g., glycosylation or disulfide bonds. Indeed, there are four predicted disulfide bonds within the CTD (Lan et al., 2020). Although *V. cholerae* and ETEC encode periplasmic enzymes for catalyzing disulfide bond formation and isomerization, the incorrect formation of disulfide bonds cannot be excluded. Two of the four disulfide bonds form between sequential, closely spaced cysteines (C336-C361 and C480-C488) are therefore likely to form correctly, while the remaining two (C379-C432 and C391-C525) are non-sequential. To reduce protein instability due to misfolding, 26 amino acids from the C-terminal end of the RBD were excluded from the LOR fusion protein, which consequently lacked the C525. In addition, the Spike protein is heavily glycosylated with two potential N-glycosylation sites (N331 and N343) in the RBD (Watanabe et al., 2020; Sanda et al., 2021). Notably, the lack of glycosylation in our bacterial system did not seem to affect the generation of a protective immune response. This could be attributed to their localization outside of the RBM, likely being the most important target antigen for neutralizing antibodies. Lack of such post-translational modifications may even be advantageous, since glycosylation of viral envelope proteins can interfere with the ability of the host to raise an adaptive immune response (Baum and Cobb, 2017).

The intranasal administration route could have advantages compared to the currently approved vaccines relying on intramuscular injections. Consistent with previous observations, intranasal application of OMVs results in robust IgG and IgA titers representing mucosal and systemic immune responses, which may act synergistically for protection (Schild et al., 2008, 2009; Bishop et al., 2010; Roier et al., 2012, 2013; Leitner et al., 2013, 2015). Vaccine development against SARS-CoV-2 has mainly focused on injections, which predominantly induce an IgG and potentially cytotoxic T cell response but lack a mucosal immune response (Russell et al., 2020; Vabret et al., 2020). However, SARS-CoV-2 is a mucosal pathogen of the nasopharynx and respiratory tract. It is becoming increasingly evident that a mucosal IgA response could be valuable for protection against SARS-CoV-2, vaccine efficacy and clearance of the virus from the infection site (Quinti et al., 2021; Wang et al., 2021). Indeed, IgA antibodies have been shown to neutralize the virus *via* binding to the RBD of SARS-CoV-2 (Ma et al., 2020; Yu et al., 2020; Sterlin et al., 2021). Based on the low secretory IgA titers in fecal pellets, lung lavage might have been a better option to analyze mucosal immune responses and should be preferred in future studies. It must be noted that lung lavage was not approved by our current animal protocol and is an endpoint assay requiring killing of the mice, while fecal pellet extraction allowed us to maintain the animals and do temporal studies, i.e., collection on days 42 and 60. Thus, we cannot exclude that other administration routes, e.g., subcutaneous, intramuscular or oral, or combinations thereof would improve the immunization efficacy, which also needs to be addressed in future comparative studies.

Several widely used vaccines have demonstrated high protective efficacy against COVID-19. However, knowledge on the longevity of antibody responses to current vaccines remains limited and additional booster vaccinations have already started in some countries, while several areas in the world do not even have sufficient access to vaccines yet. For example, less than 3% of the population are currently vaccinated on the African continent (CDC, 2021). Thus, there is still demand for development of additional vaccines to increase production volumes, facilitate accessibility as well as broaden and strengthen the immune response. In contrast to intramuscular injections used for currently approved vaccines, the non-invasive intranasal application allows an easy administration without the requirement of sterile equipment, e.g., syringes and needles. Moreover, OMV vaccine lots have been demonstrated to be stable for months even at room temperature and do not require stabilization additives (Vyssokikh et al., 2002; Bishop et al., 2010). OMVs also have advantages in terms of vaccine production. In general, donor bacteria are easy to cultivate and replicate fast. There are defined mutations, e.g., deletion of *ompA*, that result in hypervesiculating strains (Song et al., 2008; Schwechheimer and Kuehn, 2015), which in this study increased the yield of OMVs by approximately fivefold. Based on the yields presented herein, approximately 8–10 mg protein equivalent of OMVs can be isolated per 1 L of culture volume. Using a three dose regimen with 25 μg OMVs (protein equivalent) per immunization, the OMVs isolated from 1 L last for more than 100 animals. Additionally, OMVs are non-replicative and their isolation does not require treatment with inactivating agents, thus preserving the native state of antigens. These features are advantageous for mass vaccination campaigns in less developed areas of the world and could be executed even without trained health care staff.

Not surprisingly, a significant amount of research has been recently dedicated to OMV-based vaccines, including the FDA-approved vesicle-based vaccine against meningitis serogroup B (Holst et al., 2009; Caron et al., 2011; Esposito and Principi, 2014; Gandhi et al., 2016). In comparison to currently approved mRNA-based vaccines against SARS-CoV-2, an OMV-based vaccine candidate warrants easier storage and administration, which could permit worldwide use even in areas with low infrastructure and limited access to cold chain devices and medical equipment.

## Data Availability Statement

The original contributions presented in the study are included in the article/Supplementary Material, further inquiries can be directed to the corresponding author/s.

## Ethics Statement

The animal study was reviewed and approved by Federal Ministry BMBWF (protocol: 39/12/75ex2017/18).

## Author Contributions

SS and AC designed the study. All authors performed the experiments and/or the analysis, contributed to the discussion and data evaluation, and wrote the manuscript.

## Conflict of Interest

The authors declare that the research was conducted in the absence of any commercial or financial relationships that could be construed as a potential conflict of interest.

## Publisher’s Note

All claims expressed in this article are solely those of the authors and do not necessarily represent those of their affiliated organizations, or those of the publisher, the editors and the reviewers. Any product that may be evaluated in this article, or claim that may be made by its manufacturer, is not guaranteed or endorsed by the publisher.
