# Supplementary material for: An Intranasal Vaccine Based on Outer Membrane Vesicles Against SARS-CoV-2
Source: Front Microbiol. 2021 Nov 5;12:752739. doi: 10.3389/fmicb.2021.752739 (PMC8602898; doi:10.3389/fmicb.2021.752739)
Supplement: Supplementary file 1 [file Data_Sheet_1.PDF]

# *Supplementary Material*

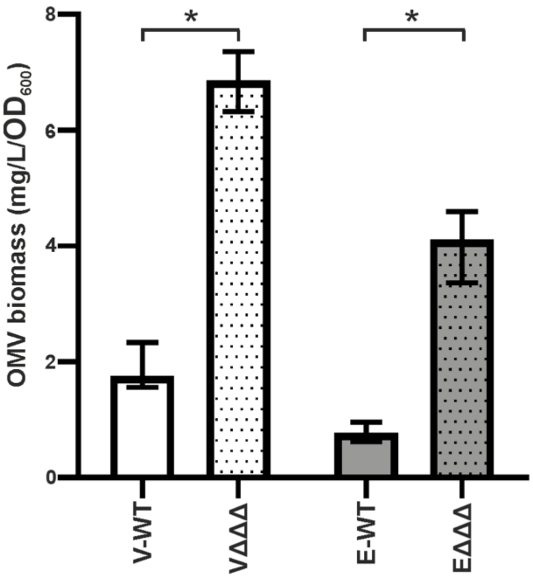


**Supplemental Figure S1. Genetically engineered detoxified triple mutants show increased vesiculation compared to the parental wild type strain.** OMV quantification (Bradford) for V-WT, VΔΔΔ, E-WT and EΔΔΔ isolated from cultures grown for 8 h at 37°C with aeration. Shown is median ± IQR ($n \geq 4$; **P* < 0.05).

# A

tca gcg tat aac tct cga caa taa tca taa aga aaa a**gg tac c**gc tag cag gag gta gaa

ttc atg aac aag atg tta atc gca gca gca gcg tct tcc gta ctt cta cta gcg ggt tgc
M N K M L I A A A A S S V L L L A G C
gcc tct ggt cct gat gaa gct acc ggt atc gta ggc gcg ttt ctg ggt tac caa gca aac
A S G P D E A T G I V G A F L G Y Q A N
aaa tgg ctc tct ctg gaa gca ggc tac gac tac tta ggt aag ttt act gct gct gga ttg
K W L S L E A G Y D Y L G K F T A A G L
aat gat gag aag gta caa gcg gta acg cta gca cca aaa ctc agc att cct ctg acc gaa
N D E K V Q A V T L A P K L S I P L T E
ggc att gcg ctg tac ggt aaa gtg ggt ggt gct tat gtc gat tac ggc agc aag gac gac
G I A L Y G K V G G A Y V D Y G S K D D
tac tca tac cta ggt gca gca ggc cta gag ttc aac act aac cac aat gtc act atg cgt
Y S Y L G A A G L E F N T N H N V T M R
ttg gaa tac caa aac ctg act gac att aat aac gac atc gta cgt gca cgc gca ggc atc
L E Y Q N L T D I N N D I V R A R A G I
ccg ggt cgc gta caa ccc acc gaa agt atc gtc cgt ttt ccc aat att acc aat ttg tgt
P G R V Q P T E S I V R F P N I T N L C
ccc ttc ggc gag gtg ttc aac gcc acg cgt ttt gca tct gta tat gca tgg aac cgc aaa
P F G E V F N A T R F A S V Y A W N R K
cgt att tcg aat tgt gtg gcg gat tat agc gtt ctc tat aat tcg gcc agt ttc tca act
R I S N C V A D Y S V L Y N S A S F S T
ttt aag tgt tat ggt gta tca cct aca aaa ctc aat gat ttg tgt ttt aca aat gtt tat
F K C Y G V S P T K L N D L C F T N V Y
gct gac agc ttc gta atc cgc ggg gat gag gtc cgc cag atc gca cca gga cag acc ggg
A D S F V I R G D E V R Q I A P G Q T G
aaa atc gcc gac tat aac tat aag ctc cct gat gat ttc act ggg tgc gta atc gcc tgg
K I A D Y N Y K L P D D F T G C V I A W
aac agc aat aat ctt gat tca aag gtt ggt ggc aat tac aac tac ctg tat cgt ttg ttc
N S N N L D S K V G G N Y N Y L Y R L F
cgt aag tca aac ttg aaa ccc ttt gag cgt gat att tct acg gag att tat caa gca ggt
R K S N L K P F E R D I S T E I Y Q A G
agt aca ccg tgc aac ggt gtt gag ggg ttc aat tgc tat ttt cca ctt caa tca tac ggg
S T P C N G V E G F N C Y F P L Q S Y G
ttt caa ccg acc aac ggc gtc ggt tat caa ccg tat cgt gtg gtc gtt ttg agc gac tac
F Q P T N G V G Y Q P Y R V V V L S D Y
aag gac gac gat gac aaa taa **gga tcc**
K D D D D K *

# B

tat cgg tag agt taa tat tga gca gat ccc ccg gtg aag gat tta acc gtg tta tct cgt

tgg aga tat tca tgg tgt att ttg gat gat aac g**gg tac c**gc tag cag gag gta gaa ttc

atg aaa gct act aaa ctg gta ctg ggc gcg gta atc ctg ggt tct act ctg ctg gca ggt
M K A T K L V L G A V I L G S T L L A G
tgc tcc agc aac gct aaa atc gat cag ggt atc ggc gct ggt gct ttt ggt ggt tac cag
C S S N A K I D Q G I G A G A F G G Y Q
gtt aac ccg tat gtt ggc ttt gaa atg ggt tac gac tgg tta ggt cgt atg ccg tac aaa
V N P Y V G F E M G Y D W L G R M P Y K
ggc agc gtt gaa aac ggt gca tac aaa gct cag ggc gtt caa ctg acc gct aaa ctg ggt
G S V E N G A Y K A Q G V Q L T A K L G
tac cca atc act gac gac ctg gac atc tac act cgt ctg ggt ggc atg gta tgg cgt gca
Y P I T D D L D I Y T R L G G M V W R A
gac act aaa tcc aac gtt tat ggt aaa aac cac gac acc ggc gtt tct ccg gtc ttc gct
D T K S N V Y G K N H D T G V S P V F A
ggc ggt gtt gag tac gcg atc act cct gaa atc gct acc cgt ctg gaa tac cag tgg acc
G G V E Y A I T P E I A T R L E Y Q W T
aac aac atc ggt gac gca cac acc atc ggc act cgt ccg gac aac ggc atc ccg ggt cgc
N N I G D A H T I G T R P D N G I P G R
gtg caa cct acc gag tct att gtg cgt ttc ccc aat atc aca aat ttg tgc ccc ttt ggt
V Q P T E S I V R F P N I T N L C P F G
gag gtg ttc aac gca acg cgt ttc gcc agc gtg tac gcg tgg aac cgt aag cgt att tcg
E V F N A T R F A S V Y A W N R K R I S
aac tgt gtg gca gat tat agc gta ttg tac aac tcc gct tct ttt agc act ttt aaa tgc
N C V A D Y S V L Y N S A S F S T F K C
tac ggg gtc agt cca acc aag tta aat gac ctg tgt ttc acc aat gtt tat gcg gac tct
Y G V S P T K L N D L C F T N V Y A D S
ttt gta atc cgt ggg gat gag gtt cgt cag att gca cct gga cag act ggg aaa att gcg
F V I R G D E V R Q I A P G Q T G K I A
gac tac aac tac aag ttg ccg gac gac ttt acc gga tgc gta att gcg tgg aac tct aac
D Y N Y K L P D D F T G C V I A W N S N
aac ctg gat tcg aag gtc ggc ggc aat tat aat tac ctg tac cgc ttg ttt cgc aaa tcg
N L D S K V G G N Y N Y L Y R L F R K S
aac ttg aaa ccc ttt gaa cgc gac atc tcg act gag atc tac caa gcc ggc agc act ccg
N L K P F E R D I S T E I Y Q A G S T P
tgc aac ggt gtt gag ggt ttc aac tgt tat ttc cca ctg caa tca tac ggg ttc cag cct
C N G V E G F N C Y F P L Q S Y G F Q P
acc aat ggt gtt gga tac cag cct tac cgt gtc gtt gtc ctg tct gac tac aag gac gac
T N G V G Y Q P Y R V V V L S D Y K D D
gat gac aaa taa **gga tcc**
D D K *

**Supplemental Figure S2. DNA and amino acid sequences of the codon-optimized Lpp-OmpA-RBD (LOR) fusion constructs for *V. cholerae* (A) and *E. coli* (B).** Shown are the in silico designed, codon-optimized DNA sequences synthesized and subcloned into the standard vector system pMK by the GeneArt Gene Synthesis platform (ThermoFisher Scientific). The corresponding amino acid sequences (if applicable) are provided in 1-letter code centered below each nucleotide triplet. Original sequence information from *V. cholerae* N16961, ETEC H10407 or SARS-CoV-2 (isolate Wuhan Hu-1) was retrieved from KEGG or NCBI databases (Kanehisa and Goto, 2000;Brister et al., 2015). Relevant features are highlighted as follows: underlined: binding of the oligonucleotides for amplification (LOR_E_1 and LOR_E_BamHI_2 as well as LOR_V_1 and LOR_V_BamHI_2); light grey and bold: restriction sites KpnI and BamHI for sublconing into pMMB67EH; dark grey: Shine-Dalgarno sequence; light blue: Lpp fragment; green: linker; red: OmpA fragment; yellow: SARS-CoV-2 RBD fragment; pink: FLAG-tag.

Brister, J.R., Ako-Adjei, D., Bao, Y., and Blinkova, O. (2015). NCBI viral genomes resource. *Nucleic Acids Res* 43**,** D571-577.

Kanehisa, M., and Goto, S. (2000). KEGG: kyoto encyclopedia of genes and genomes. *Nucleic Acids Res* 28**,** 27-30.

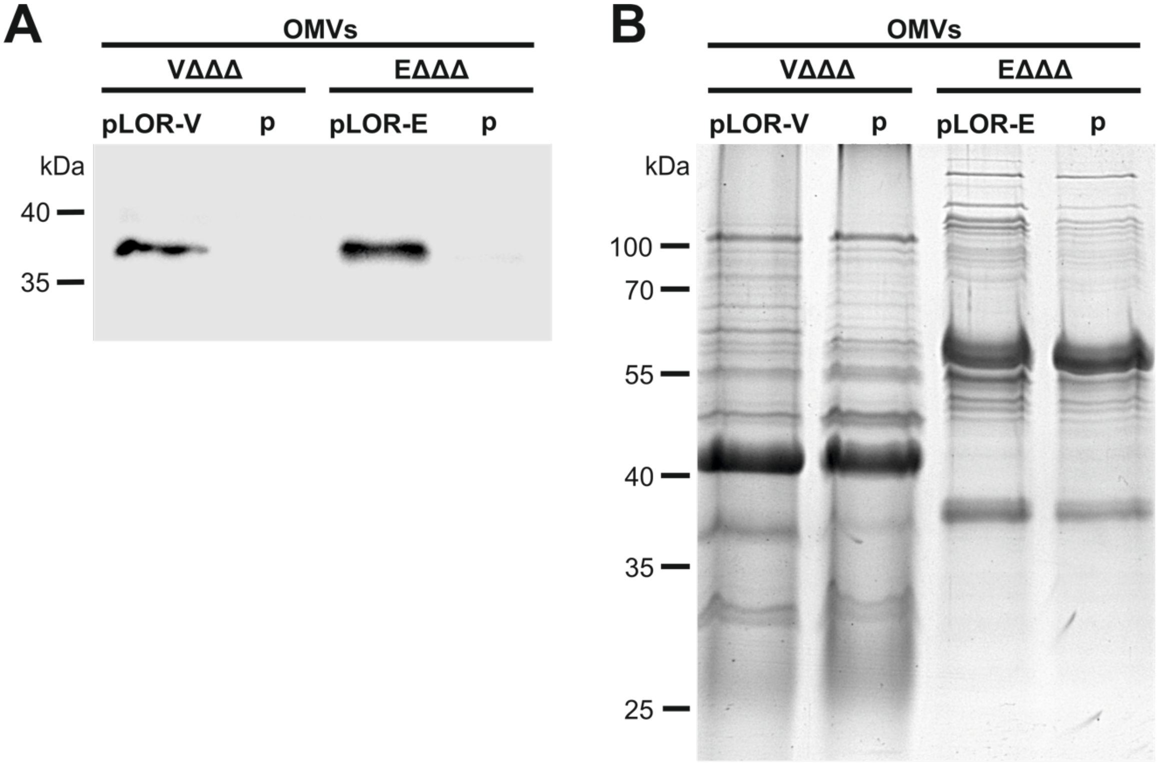


**Supplemental Figure S3. Additional characterization of LOR decoration of OMVs. (A)** Shown is a representative immunoblot detecting the LOR fusion protein in OMVs derived from VΔΔΔ pLOR-V and VΔΔΔ p, EΔΔΔ pLOR-E and EΔΔΔ p. The commercially available anti-FLAG antisera specifically detecting the C-terminal FLAG-tag of the LOR fusion protein was used for this immunoblot. (B) SDS-PAGE and Kang staining was executed in parallel with the same samples used for the immunoblot analyses provided in Fig. 1B and S3A and serves as a loading control. Molecular mass standards (PageRuler Prestained Protein Ladder – Thermo Fisher Scientific) are indicated on the left.

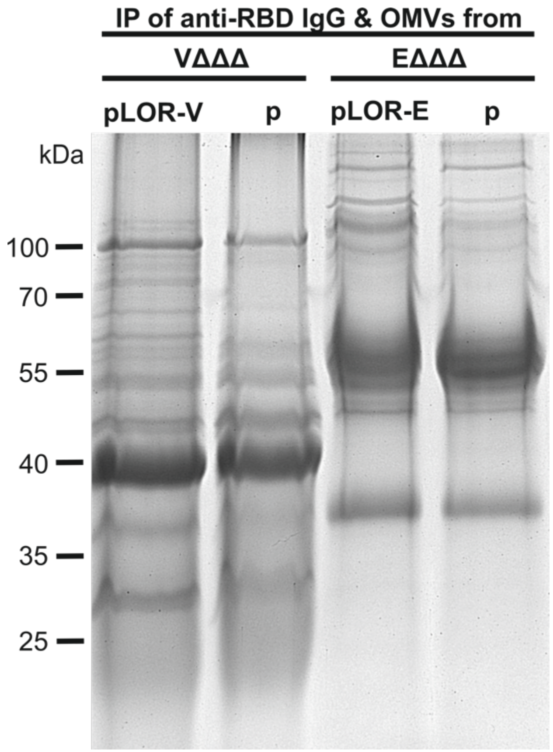


**Supplemental Figure S4. Protein profiles of the immunoprecipitation (IP).** SDS-PAGE and Kang staining was executed in parallel with the same samples used for the immunoblot analyses provided in Fig. 1C and serves as a loading control. Molecular mass standards (PageRuler Prestained Protein Ladder – Thermo Fisher Scientific) are indicated on the left.

| OMVs derived from | Diameter [nm] | | Biomass [mg/L/$OD_{600}$] | |
|---|---|---|---|---|
| | Mean median (min-max) | Mode median (min-max) | Bradford median (min-max) | Purpald median (min-max) |
| V-WT | 105.9 (96.54-110.2) | 135.5 (120.9-144.5) | 1.76 (1.36-2.59) | 0.39 (0.31-0.46) |
| VΔΔΔ | 117.4 (115.2-123.2) | 145.8 (138.0-153.4) | 6.87 (4.79-8.89) | 0.63 (0.59-0.66) |
| VΔΔΔ pLOR-V | 105.1 (101.8-105.7) | 126.3 (120.1-136.0) | 5.28 (4.70-5.68) | 0.45 (0.39-0.50) |
| VΔΔΔ p | 106.6 (103.0-112.1) | 130.1 (123.1-138.4) | 5.37 (2.88-5.92) | 0.41 (0.37-0.49) |
| E-WT | 155.6 (137.8-182.0) | 219.5 (168.3-280.5) | 0.78 (0.53-1.34) | 0.15 (0.14-0.16) |
| EΔΔΔ | 131.6 (128.5-139.9) | 166.0 (158.8-176.1) | 4.12 (3.61-4.48) | 0.60 (0.53-0.75) |
| EΔΔΔ pLOR-E | 109.3 (105.1-113.4) | 139.0 (127.8-167.8) | 4.16 (3.86-4.49) | 0.24 (0.22-0.29) |
| EΔΔΔ p | 111.1 (101.4-118.1) | 135.9 (124.9-159.0) | 3.57 (3.53-4.04) | 0.28 (0.20-0.25) |

**Supplemental Figure S5. Size distribution and biomass quantification of OMVs used in this study.** Mean and mode OMV diameter sizes were measured by Zetasizer. Biomass quantification of OMV preparations derived from equivalent $OD_{600}$ units of the respective cultures were analyzed for total protein (Bradford) or lipopolysaccharide (Purpald). Median with maximum and minimum values are shown ($n \geq 5$ biological replicates).

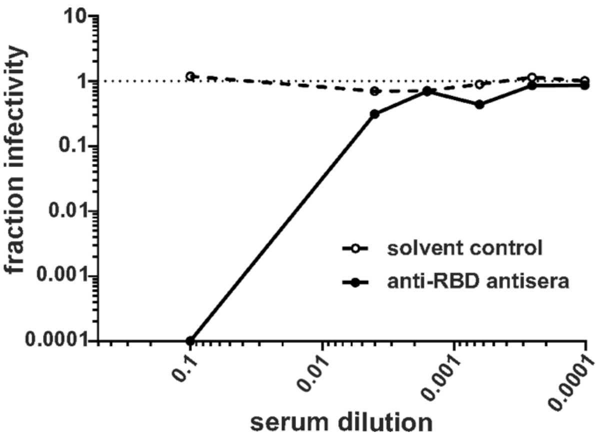


**Supplemental Figure S6. Controls for the neutralization assay.** Shown is the median fraction of infectivity using a Spike-pseudotyped lentivirus neutralization assay in combination with commercially available anti-RBD antisera (PA5-114451, ThermoFisher Scientific). Starting with a 1:10 dilution, 2.5-fold serial dilutions of the anti-RBD antisera were tested in duplicate to proof functionality of the assay. Appropriate dilutions of PBS served as solvent (negative) control. The limit of detection in these assays was a fraction of infectivity = 0.0001.
